# The influence of cerebellum on visual selective attention in patients with multiple lacunar cerebral infarction and its neuromodulatory mechanisms

**DOI:** 10.3389/fnhum.2024.1380739

**Published:** 2024-04-23

**Authors:** Xiaodong Yuan, Liqin Duan, Ya Ou, Qirong Ling, Jing Wang, Jian Zhang, Lingyun Cao, Hongchun Qian, Pingshu Zhang

**Affiliations:** ^1^Department of Neurology, Kailuan General Hospital, North China University of Technology, Tangshan, Hebei, China; ^2^Key Laboratory of Neurobiological Function in Hebei Province, Tangshan, Hebei, China

**Keywords:** multiple lacunar cerebral infarction, cerebellar infarction, event-related potentials, source reconstruction, visual selective attention function

## Abstract

**Objective:**

This study aims to investigate the influence of the cerebellum on visual selective attention function and its neuromodulatory mechanism in patients with multiple lacunar cerebral infarction (MLCI).

**Methods:**

A retrospective analysis was conducted on 210 patients admitted with MLCI from January 2016 to May 2022. Analyzed the electrophysiological characteristics of the P3a and P3b components of vision in both groups, as well as source reconstruction simulations of dipole activation in the brains of the two groups, and analyzed the brain regions with differences in activation strength between the two groups.

**Results:**

This study found that there was no significant difference in peak amplitude between the two groups, but compared with the control group, the peak latency of the case group was significantly prolonged. Specifically, the P3a peak latency induced by the novel stimulus was longer than that induced by the target stimulus P3b peak latency. Source reconstruction results showed decreased and increased activation in several brain regions in the case group compared to the control group.

**Conclusion:**

The study suggests that the impairment of distracted attention capture is more pronounced in patients with MLCI. The cerebellum indirectly influences the ventral and dorsal frontoparietal attention networks by modulating the levels of excitation and inhibition within the cerebral cortex of the attention network. This may represent a potential mechanism through which the cerebellum regulates visual selective attention information in MLCI patients.

## 1 Introduction

Multiple lacunar cerebral infarction (MLCI) is the prevalent form of ischemic stroke, constituting 25% of all ischemic strokes (Gore et al., 2023). MLCI is frequently underestimated by clinicians due to its subtle onset and challenges in detection through imaging. However, MLCI is predisposed to cognitive dysfunction and may even lead to the development of vascular dementia, impacting the quality of life for patients, placing a burden on families, and gradually posing a medical challenge.

Cognitive impairment following cerebellar infarction is substantiated by an expanding body of clinical and research evidence (Mehta et al., 2020; Erdal et al., 2021; Gok-Dursun et al., 2021). Originating from the anterior part of the rhombencephalon, the cerebellum develops earlier than the cerebrum during embryonic development’s early stages (Keefe and Nowakowski, 2020). Notably, some studies indicate that the cerebellar cortex is more condensed than the cerebral cortex, constituting approximately 78% of the neocortex’s surface area in the human brain. This suggests a pivotal role of the cerebellum in the evolution of human cognition (Sereno et al., 2020). Our study anticipates delving into the impact of the cerebellum on cognitive function in patients with MLCI and its underlying mechanisms. This exploration aims to unveil novel pathways for treating MLCI.

Previous investigations into cognitive functioning have commonly utilized neuropsychological scales, functional magnetic resonance imaging, transcranial magnetic electrical stimulation, and biochemical tests (Bolcekova et al., 2017; Nam et al., 2020; Ni et al., 2023; Yao et al., 2023). However, some of these research methods exhibit high subjectivity, and others are limited to capturing only a single dimension of cognition. Event-related potentials (ERPs) serve as time-locked, non-invasive, straightforward, and inexpensive techniques capable of recording neuronal activity on millisecond timescales. They represent objective methods employed to study the neurophysiological activity of cognitive functions (Helfrich and Knight, 2019). The P300 component stands out as one of the classical components in ERPs employed for investigating cognitive functions. It typically encompasses two subcomponents, P3a and P3b. These components reflect different aspects of attentional functioning, with the P3a component indicating the capture of distracting information and being associated with frontal dopaminergic activity. In contrast, the P3b component reflects attention to task-relevant information and is linked to memory and descending adrenergic parietal activity (Polich, 2007; Masson and Bidet-Caulet, 2019; Riby et al., 2023). Previous studies, including those examining cerebellar cognitive function (Rodriguez-Labrada et al., 2019; Nanda et al., 2023), have demonstrated the vital role of the P300 component in uncovering the involvement of the cerebellum in cognitive functioning.

The primary aim of source reconstruction is to provide insights into both the origin of the power source and its anatomical location, facilitating the interpretation of measured bioelectrical signals. Paitel and Nielson (2023) employed source reconstruction techniques to investigate cerebellar activation in an elderly population, distinguishing carriers and non-carriers of the ε4 allele—a gene highly associated with Alzheimer’s disease—during the P300 component. In our study, we delved into the neurophysiological impact of cerebellar infarction on visual P300 neurophysiology in patients with MLCI using ERPs. Utilizing ERP fusion-averaged magnetic resonance imaging datasets from the Montreal Neurological Institute for source reconstruction, our objective was to unveil the underlying neural mechanisms of cerebellar infarction on visually selective attentional function in MLCI patients. Our hypothesis posited that the cerebellum is intricately involved in visual selective attention function in MLCI patients, and that cerebellar infarction exacerbates dysfunction in visual selective attention within this patient cohort.

## 2 Materials and methods

### 2.1 Participants

This study conducted a retrospective analysis of patients admitted to the Department of Neurology at Kailuan General Hospital from January 2016 to May 2022, applying specific inclusion and exclusion criteria:

Inclusion criteria: (1) age 18–80 years old; (2) Brain magnetic resonance imaging (MRI) results suggest that the infarct area < 20 mm; (3) MRI scan of the case group showed cerebellar infarction, the number of cerebral infarction areas ≥ 2, MRI scan of the control group showed no cerebellar infarction, the number of cerebral infarction areas ≥ 2; (4) National Institute of Health stroke scale (NIHSS) score ≤ 4; (5) no visual, auditory disorders; (6) no serious neuropsychiatric diseases; (7) no history of craniocerebral trauma; (8) can cooperate with the completion of the experiment independently; (9) has signed the informed consent form.

Exclusion criteria: (1) age < 18 years old or > 80 years old; (2) the presence of cerebellar neurodegenerative diseases; (3) the presence of visual or auditory impairments, the presence of severe comprehension disorders, the presence of severe neuropsychiatric disorders, the presence of a history of cranio-cerebral trauma, and inability to cooperate to complete the experiment independently; (4) NIHSS score > 4.

**FIGURE 1:**
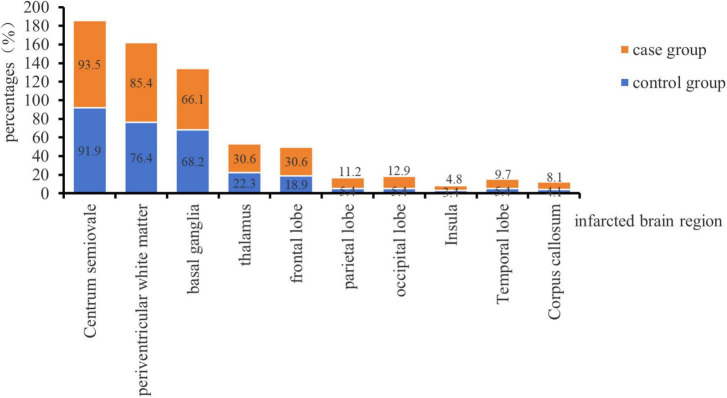
Distribution of infarcted brain regions in the two groups.

A total of 210 patients with cerebral infarction were included in this study, comprising 62 patients with cerebellar infarction combined with bilateral MLCI as the case group and 148 patients with bilateral MLCI as the control group Figure 1 demonstrates the proportion of the number of cerebral infarcted brain regions in the two groups, and the difference in the number of individual brain regions between the two groups was not statistically significant (*P* < 0.05). Additionally, there were a total of 25 left cerebellar infarcts, 22 right cerebellar infarcts, and 15 bilateral cerebellar infarcts in the case group. The age distribution of the case group ranged from 38 to 77 years, consisting of 45 males and 17 females. The control group exhibited an age distribution of 38 to 77 years, with 104 males and 44 females. This study received approval from the Ethics Committee of Kailuan General Hospital (No. 2022005).

### 2.2 Experimental paradigm design

**FIGURE 2:**
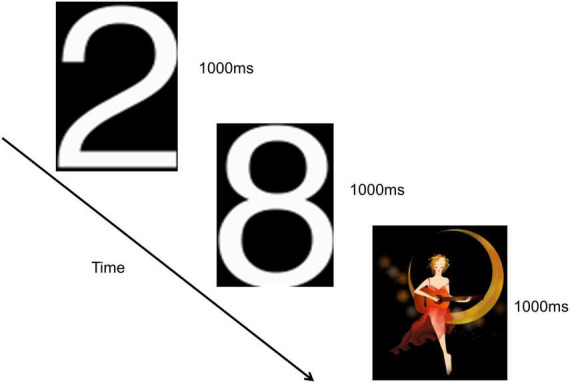
Visual P300 experimental paradigm.

This study employed the classical oddball paradigm visual P300, as depicted in Figure 2. Visual stimuli were presented using E-prime software, and subjects were situated in a shielded room, isolated from external disturbances, maintaining a quiet and suitable temperature. The distance between the subjects’ eyes and the screen was less than 1 m. Before the formal experiment, a pre-exercise was conducted. During this pre-exercise, a picture was randomly and rapidly displayed on the computer screen. Subjects were instructed to place their right hand on the mouse in advance and click the left mouse button when the picture of number “2” appeared. This process was repeated until subjects successfully mastered the operation, signaling the commencement of the formal experiment.

The number “2” pictures were presented in white, while the “moon girl” pictures were in color, both against a black background. The “moon girl” picture served as a novel stimulus, measuring 294 × 338 pixels, eliciting a visual P3a potential. The number “2” picture acted as a target stimulus, measuring 113 × 175 pixels, evoking a visual P3b potential. The number “8” picture served as a non-target stimulus, measuring 113 × 171 pixels. The novel stimulus was randomly presented 30 times, the target stimulus was randomly 30 times, and the number “8” picture was randomly appeared 170 times. Deviant stimuli comprised both the novel and target stimuli. Each picture was displayed for 1000 ms without intervals between presentations, and the entire experiment lasted approximately 4 min.

### 2.3 EEG data acquisition

In this study, the paradigms presented by the E-prime software were interfaced with the Curry7 software through an external connection device. The Curry7 software recorded the electroencephalogram (EEG) and automatically annotated the corresponding time of paradigm presentation. The US Neuroscan 64 conductive electrode cap was used to record EEG, and the electrode position of the 10–10 system was extended according to the international 10–20 system. Electrodes were positioned 1 cm below the upper left eye and 1 cm from the outer canthus of both eyes to capture horizontal and vertical ophthalmoplegia. Electrode resistance was maintained below 5 KΩ, and the sampling rate was set at 1,000 Hz bilateral mastoids were used as reference electrodes.

### 2.4 Pre-processing of EEG data

The raw EEG data underwent preprocessing using Curry 7 software, involving the following steps: (1) re-referencing with M_1_ and M_2_ electrodes as the reference points; (2) band-pass filtering with a range of 1–10 Hz and an intensity of 2–8 Hz; (3) baseline correction, selecting −200 to 0 ms for correction; (4) de-artifacting, with an amplitude cut-off of −200 μV, and removal of ocular artifacts during blinking, occurring between −200 to 500 ms, with a total duration of 700 ms; (5) removal of invalid leads; (6) The EEG evoked by stimuli of the same class was overlaid and averaged with a time window of −200 to 700 ms; (7) repeated baseline correction; and (8) saving of ERP data evoked by corresponding stimuli for visual P3a and visual P3b, respectively.

### 2.5 Analysis of event-related potentials

The pre-processed P3a and P3b components underwent segmentation based on time intervals, and peak amplitude and peak latency were subsequently identified. For the analysis in this study, ERP waveforms recorded from three electrodes—FZ (central frontal), CZ (central), and PZ (central parietal)—were primarily chosen due to their reduced susceptibility to electromyography and ophthalmoscopy interference. Averaged waveform plots illustrating the distribution of P300 component wave amplitude and latency were generated using Matlab software along with the eeglab plug-in.

### 2.6 Source reconstruction analysis

The precision of source reconstruction can be enhanced by elevating the signal-to-noise ratio (SNR) of the data, and a SNR of 10 or more indicates more reliable source reconstruction results. In this study, the pre-processed ERPs underwent additional steps, including M_1_ and M_2_ re-referencing, baseline correction, and filtering, aimed at improving the SNR of the data. The mean global field power (MGFP) represents the cumulative electromagnetic activity of EEG signals within a specific time frame, providing a measure of the overall electrical activity intensity in the brain. The P3a and P3b components of all electrodes can be divided according to MGFP.

#### 2.6.1 Principle component and independent component analyses

Principal components analysis (PCA) and independent components analysis (ICA) were conducted using Curry7 software. Initially, PCA is employed to reduce the dimensionality of the high-dimensional data and transform the original data into a set of linearly independent data in each dimension. This transformation is utilized to extract the primary feature components of the data. Subsequently, ICA is applied to recover the mixed signals within each feature component as independent original signals, generating waveforms that are as independent as possible. Curry7 software presents the MGFP after both PCA and ICA, providing insight into the level of brain activity during different P300 tasks. Lastly, spatio-temporal filtering of the measurements over selected time frames is implemented to eliminate noisy or irrelevant components, thereby better isolating and highlighting the components of interest.

#### 2.6.2 The boundary element method for creating volume conductor models

In this study, we used an average T_1_-weighted MRI image dataset to create a volume conductor model that conforms to the anatomical structure of the real brain. The average T_1_-weighted MRI data, generated by the Montreal Neurological Institute, were encapsulated within the Curry7 software. The boundary element method employed a fixed matrix (consisting of nodes and triangular meshes) to establish a standardized volume conductor model, following the methodology outlined by Fuchs (Fuchs et al., 2002). The construction of the high-resolution volume conductor model involved 5,413 nodes (skin layer 1679, outer cranial bone layer 1624, inner cranial bone layer 2110) and 10814 triangular meshes (skin layer 3354, outer cranial bone layer 3244, inner cranial bone layer 4216). The conductivity of the skin layer, inner cranial bone layer, and outer cranial bone layer was 0.33 s/m, 0.0042 s/m, and 0.33 s/m, respectively. The isolated problem method was employed to enhance the model accuracy. All procedures were executed using Curry7 software.

#### 2.6.3 Dipole source reconstruction

The simplest dipole source model is the single equivalent current dipole, with the moving dipole serving as an extension. In this study, the source reconstruction utilized moving dipoles to more accurately simulate the propagation sources and paths during cognitive processing in the brain. The positions of the dipoles were iteratively optimized until a well-fitted dipole adequately explained the data. The number of dipoles was determined by the count of SNRs greater than 1 after principal component analysis. In this study, the automatic mode was chosen to conduct a coarse deviation scan across a predefined set of positions, with the best position identified and used as a seed. The seed distance represents the maximum distance from the best-fit position (seed point) to the starting position in the multidimensional nonlinear Nelder-Mead fitting algorithm, typically set at 200 mm by default. To prevent strong opposite (canceling) source configurations, a minimum distance of 20 mm between dipole positions was selected. The quality of the fit was assessed based on variance results, with a fit considered good if the variance reached 90% or more. Parameters were automatically generated by Curry 7 software upon completion of the parameter settings.

### 2.7 Statistical analyses

All statistical analyses were performed using SPSS 27.0. The Mann–Whitney *U* test was employed to compare the age between the two groups. Pearson’s test was utilized for the comparison of gender, education, and risk factors between the two groups. Repeated measures multifactor ANOVA was applied to compare peak amplitude and peak latency. For data obtained from the source reconstruction of brain interval activation strengths that adhered to a normal distribution, the *t*-test was used to compare the mean values of measurement data conforming to normal distribution between the two groups. Do not conform to the normal distribution of measurement data comparison between the two groups using nonparametric the Mann–Whitney *U* test. Descriptive statistics were presented as “mean ± standard” deviation for continuous variables and as percentages for categorical variables. The significance level (α) was set at 0.05.

## 3 Results

### 3.1 Comparison of demographic and risk factor information between the two groups

Table 1 presents the statistical results for age, gender, and education level in both the case and control groups. The results indicate that the comparison of amplitude, latency, and activation intensity of brain regions between the two groups was not influenced by age, gender, and education (*P* < 0.05). Table 2 displays the statistical results concerning risk factors between the two groups. The difference was not statistically significant between the two groups for smoking, drinking, diabetes, and coronary heart disease, hyperuricaemia, hyperlipidemia, hyperhomocysteinemia (*p* > 0.0.5). However, the difference in hypertensive disease (*P* < 0.05) between the two groups was statistically significant.

**TABLE 1:** Results for age, gender, and educational level in the case and control groups [$\bar{x}$ ± s, *n* (%)].

Groups		Case group (*n* = 62)	Control group (*n* = 148)	*t*/*x*^2^/*z*	*P*
Age		64.64 ± 8.23	63.10 ± 9.05	−1.384*	0.166
Gender	Male	45	104	0.113**	0.737
	Female	17	44		
Educational level	Primary and below	10 (16.1)	34 (23.0)	1.618***	0.445
	Middle school	44 (71.0)	92 (62.2)		
	High school and above	8 (12.9)	22 (14.9)		

*is *t*-value;

**is *x*^2^ value;

***denotes *z* value of rank sum test.

**TABLE 2:** Risk factor results of case group and control group *n* (%).

Groups	Case group (*n* = 62)	Control group (*n* = 148)	χ^2^	*P*
Smoking	33 (53.23)	61 (41.23)	2.549	0.110
Drinking	25 (40.32)	51 (34.46)	0.650	0.420
Diabetes	21 (33.87)	34 (22.97)	2.685	0.101
Coronary heart disease	13 (20.97)	25 (16.89)	0.490	0.484
Hypertensive disease	51 (82.26)	99 (66.89)	5.06	0.03
Hyperuricaemia	15 (24.19)	22 (14.86)	2.620	0.106
Hyperlipidemia	25 (40.32)	70 (47.30)	0.936	0.333
Hyperhomocysteinemia	33 (53.23)	79 (53.38)	0.003	0.960

### 3.2 Event-related potential P300 component amplitude and latency results

#### 3.2.1 Event-related potential P300 peak amplitude results

There was no significant difference in the main effect between groups (*F* = 2.078, *p* = 0.151, η^2^ = 0.010). However, the main effect of stimulus condition was statistically significant (*F* = 81.031, *p* < 0.001, η^2^ = 0.280), indicating that the P3a peak amplitude evoked by the novel stimulus (3.06 ± 0.13) was significantly lower (*p* < 0.001) than the P3b peak amplitude evoked by the target stimulus (4.87 ± 0.22). The main effect of electrode points was statistically significant (*F* = 20.187, *p* < 0.001, η^2^ = 0.088). Specifically, the amplitude recorded by the CZ electrode (3.93 ± 0.15) was significantly lower (*p* = 0.001) than that recorded by the FZ electrode (4.37 ± 0.19). Additionally, the amplitude recorded by the PZ electrode (3.59 ± 0.14) was significantly lower (*p* < 0.001) than that recorded by the FZ electrode (4.37 ± 0.19), and the amplitude recorded by the PZ electrode (3.59 ± 0.14) was lower (*p* < 0.001) than that recorded by the CZ electrode (3.93 ± 0.15), as detailed in Table 3.

**TABLE 3:** Results of main and interaction effects of P300 peak amplitude in groups, stimulus conditions and electrode points.

		Peak amplitude	*F*	Freedom	*P*	η^2^
Groups	Case group (*n* = 62)	3.75 ± 0.25	2.078	1.00	0.15	0.010
	Control group (*n* = 148)	4.17 ± 0.16				
Stimulus conditions	Novel stimulus	3.06 ± 0.13	81.031	1.00	0	0.280
	Target stimulus	4.87 ± 0.22				
Electrode points	FZ	4.37 ± 0.19	20.187	1.40	0	0.088
	CZ	3.93 ± 0.15				
	PZ	3.59 ± 0.14				
Stimulus conditions × groups			0.619	1.00	0.43	0.003
Electrode points × groups			0.315	1.40	0.65	0.002
Stimulus conditions × electrode points			15.046	1.73	0	0.067
Stimulus conditions × electrode points × groups			0.035	1.73	0.95	0

The second-order interaction between stimulus conditions and groups did not reach statistical significance (*F* = 0.619, *p* = 0.43, η^2^ = 0.003), and the second-order interaction between electrode points and groups was also not statistically significant (*F* = 0.315, *p* = 0.65, η^2^ = 0.002). However, the second-order interaction between stimulus conditions and electrode points was statistically significant (*F* = 15.046, *p* < 0.001, η^2^ = 0.067), as shown in Table 3. Further examination of simple effects of the stimulus condition revealed that the P3a peak amplitude evoked by the novel stimulus recorded at the FZ electrode (3.73 ± 0.17) was significantly lower (*p* < 0.05) than the P3b peak amplitude evoked by the target stimulus (5.01 ± 0.27). Similarly, the P3a peak amplitude evoked by the novel stimulus recorded at the CZ electrode (3.05 ± 0.14) was significantly lower (*p* < 0.05) than the P3b peak amplitude evoked by the target stimulus (4.81 ± 0.24). Moreover, the P3a peak amplitude produced by the novel stimulus recorded at the PZ electrode (2.39 ± 0.12) was significantly lower (*p* < 0.05) than the P3b peak amplitude evoked by the target stimulus (4.79 ± 0.21), as indicated in Table 4. Regarding the difference of the simple effect of electrode points was no statistically significant in the peak amplitude of the P3b induced by the target stimulus was observed among the three electrode points FZ, CZ, and PZ (*p* > 0.05). However, the P3a peak amplitude evoked by novel stimuli recorded at the CZ electrode (3.05 ± 0.14) was significantly lower (*p* < 0.001) than that recorded at the FZ electrode (3.73 ± 0.17). Furthermore, the P3a peak amplitude evoked by novel stimuli recorded at the PZ electrode (2.39 ± 0.12) was significantly lower (*p* < 0.001) than that recorded at the FZ electrode (3.73 ± 0.17), and the P3a peak amplitude of the novel stimulus recorded by the PZ electrode (2.39 ± 0.12) was significantly lower (*p* < 0.001) than that recorded by the CZ electrode (3.05 ± 0.14), as detailed in Table 5.

**TABLE 4:** Results of simple effects of stimulus conditions in the interaction between stimulus conditions and electrode points peak amplitude.

Electrode points	Target stimulus	Novel stimulus	Average difference^a^	Average difference 95% CI	*F*	*P*	η^2^
FZ	5.01 ± 0.27	3.73 ± 0.17	1.283*	(0.79, 1.77)	26.50	0	0.11
CZ	4.81 ± 0.24	3.05 ± 0.14	1.759*	(1.28, 2.24)	52.40	0	0.20
PZ	4.79 ± 0.21	2.39 ± 0.12	2.394*	(1.99, 2.80)	137.33	0	0.40

*Indicates the significance level of the difference in average *P* < 0.05;

^a^Indicates the average peak amplitude evoked by the target stimulus minus the average peak amplitude evoked by the novel stimulus.

**TABLE 5:** Results of electrode points simple effects in the interaction between stimulus conditions and electrode points peak amplitude.

Electrode points	FZ	CZ	PZ	Average difference^a^	Average difference 95% CI	*F*	*P*	η^2^
Novel stimulus	3.73 ± 0.17	3.05 ± 0.14	2.39 ± 0.12	0.674*/1.334*/0.660*	(0.423, 0.926)/(0.957, 1.711)/(0.409, 0.911)	36.25	0	0.26
Target stimulus	5.01 ± 0.27	4.81 ± 0.24	4.79 ± 0.21	0.198/0.222/0.024	(−0.254, 0.651)/(−0.331, 0.775)/(−0.306, 0.354)	0.58	0.56	0.01

*Indicates a significance level of *P* < 0.05 for the difference in average.

^a^Indicates the average value of the peak amplitude recorded by the FZ electrode minus the average value of the peak amplitude recorded by the CZ electrode; the average value of the peak amplitude recorded by the FZ electrode minus the average value of the peak amplitude recorded by the PZ electrode; the average value of the peak amplitude recorded by the CZ electrode minus the average value of the peak amplitude recorded by the PZ electrode.

The third-order interaction among stimulus conditions, electrode points, and groups did not reach statistical significance (*F* = 0.035, *p* = 0.95, η^2^ = 0), as indicated in Table 3.

#### 3.2.2 Event-related potential P300 peak latency results

The group main effect reached statistical significance (*F* = 9.776, *p* = 0.002, η^2^ = 0.045), indicating that the P300 peak latency in the case group (481.39 ± 6.61) was longer (*p* = 0.002) than in the control group (456.79 ± 4.28). The stimulus condition main effect was also statistically significant (*F* = 87.81, *p* < 0.001, η^2^ = 0.297), suggesting that the P3a peak latency produced by the novel stimulus (499.85 ± 5.55) was longer (*p* < 0.001) than the P3b peak latency produced by the target stimulus (438.33 ± 4.67). Furthermore, the main effect of the electrode point reached statistical significance (*F* = 25.119, *p* < 0.001, η^2^ = 0.108), indicating that the latency recorded by the CZ electrode (476.08 ± 4.35) was longer (*p* < 0.001) than that recorded by the FZ electrode (454.26 ± 4.68), and the latency recorded by the PZ electrode (476.93 ± 4.34) was longer (*p* < 0.001) than the FZ electrode latency (454.26 ± 4.68). However, there was no statistically significant difference in the peak latency of the P300 recorded by the CZ and PZ electrodes (*p* = 1.00), as shown in Table 6.

**TABLE 6:** Results of main and interaction effects of P300 peak latency in groups, stimulus conditions and electrode points.

		Peak latency	*F*	Freedom	*P*	η^2^
Groups	Case group (*n* = 62)	481.39 ± 6.61	9.776	1.00	0.002	0.045
	Control group (*n* = 148)	456.79 ± 4.28				
Stimulus conditions	Novel stimulus	499.85 ± 5.55	87.81	1.00	0	0.297
	Target stimulus	438.33 ± 4.67				
Electrode points	FZ	454.26 ± 4.68	25.119	1.677	0	0.108
	CZ	476.08 ± 4.35				
	PZ	476.93 ± 4.34				
Stimulus conditions × groups			0.011	1.00	0.915	0
Electrode points × groups			1.241	1.677	0.286	0.006
Stimulus conditions × electrode points			8.952	1.816	0	0.041
Stimulus conditions × electrode points × groups			0.395	1.816	0.654	0.002

The second-order interaction between stimulus conditions and groups was not statistically significant (*F* = 0.011, *p* = 0.915, η^2^ = 0). The second-order interaction between electrode points and groups also did not reach statistical significance (*F* = 1.241, *p* = 0.286, η^2^ = 0.006). However, the second-order interaction between stimulus conditions and electrode points reached statistical significance (*F* = 8.952, *p* < 0.001, η^2^ = 0.041), as indicated in Table 6. Examining the simple effects of stimulus conditions, the P3a peak latency of the novel stimulus (477.32 ± 6.62) recorded by the FZ electrode was significantly longer (*p* < 0.001) than the P3b peak latency of the target stimulus (431.19 ± 5.52). The CZ electrode recorded a longer (*p* < 0.001) P3a peak latency for the novel stimulus (509.39 ± 6.13) than for the P3b peak latency of the target stimulus (442.77 ± 5.07). Similarly, the P3a peak latency of the novel stimulus (512.85 ± 6.3) recorded by the PZ electrode was significantly longer (*p* < 0.001) than the P3b peak latency of the target stimulus (441.02 ± 5.22), as detailed in Table 7. Considering the simple effects of electrode points, the P3b latency evoked by the target stimulus recorded at the CZ electrode (442.77 ± 5.07) was significantly longer (*p* = 0.035) than that recorded at the FZ electrode (431.19 ± 5.52). However, no statistically significant difference (*p* = 0.131) was observed between the P3b peak latency of the target stimulus recorded by the FZ electrode (431.19 ± 5.52) and that recorded by the PZ electrode (441.02 ± 5.22). Additionally, there was no statistically significant difference (*p* = 1.00) between the P3b peak latency of the target stimulus recorded by the CZ electrode (442.77 ± 5.07) and that recorded by the PZ electrode (441.02 ± 5.22). Regarding the P3a peak latency of novel stimuli, recordings by the CZ electrode (509.39 ± 6.13) were significantly longer (*p* < 0.001) than those recorded by the FZ electrode (477.32 ± 6.62). Similarly, the P3a peak latency of novel stimuli recorded by the PZ electrode (512.85 ± 6.3) was significantly longer (*p* < 0.001) than that recorded by the FZ electrode (477.32 ± 6.62). There was no statistically significant difference (*p* = 1.000) between the P3a peak latencies of novel stimuli recorded by CZ electrodes (509.39 ± 6.13) and those recorded by PZ electrodes (512.85 ± 6.3), as shown in Table 8.

**TABLE 7:** Results of simple effects of stimulus conditions in the interaction between stimulus conditions and electrode points peak latency.

Electrode points	Novel stimulus	Target stimulus	Average difference^a^	Average difference 95% CI	*F*	*p*	η^2^
FZ	477.32 ± 6.62	431.19 ± 5.52	−46.14*	(−61.53, −30.74)	34.89	0	0.14
CZ	509.39 ± 6.13	442.77 ± 5.07	−66.62*	(−80.68, −52.56)	87.27	0	0.30
PZ	512.85 ± 6.3	441.02 ± 5.22	−71.83*	(−86.93, −56.72)	87.91	0	0.30

*Denotes the significance level of the difference in means at *P* < 0.05.

^a^Indicates the average of peak latencies evoked by the target stimulus minus the average of peak latencies evoked by the novel stimulus.

**TABLE 8:** Results of electrode points simple effects in the interaction between stimulus conditions and electrode points peak latency.

Electrode points	FZ	CZ	PZ	Average difference^a^	Average difference 95% CI	*F*	*P*	η^2^
Novel stimulus	477.32 ± 6.62	509.39 ± 6.13	512.85 ± 6.3	−32.07*/−35.52*/−3.46	(−43.60, −20.54)/(−50.89, −20.16)/(−15.08, 8.17)	23.58	0	0.19
Target stimulus	431.19 ± 5.52	442.77 ± 5.07	441.02 ± 5.22	−11.58*/−9.83/1.75	(−22.55, −0.61)/(−21.52, 1.86)/(−5.91, 9.41)	3.23	0.041	0.03

*Indicates a significance level of *P* < 0.05 for differences in means.

^a^Indicates the average value of peak latency recorded by the FZ electrode minus the mean value of peak latency recorded by the CZ electrode; the average value of peak latency recorded by the FZ electrode minus the mean value of peak latency recorded by the PZ electrode; the average value of peak latency recorded by the CZ electrode minus the mean value of peak latency recorded by the PZ electrode, respectively.

There was no statistically significant third-order interaction among stimulus conditions, electrode points, and groups (*F* = 0.395, *p* = 0.654, η^2^ = 0.002).

#### 3.2.3 Event-related potential group averaging plot

**FIGURE 3:**
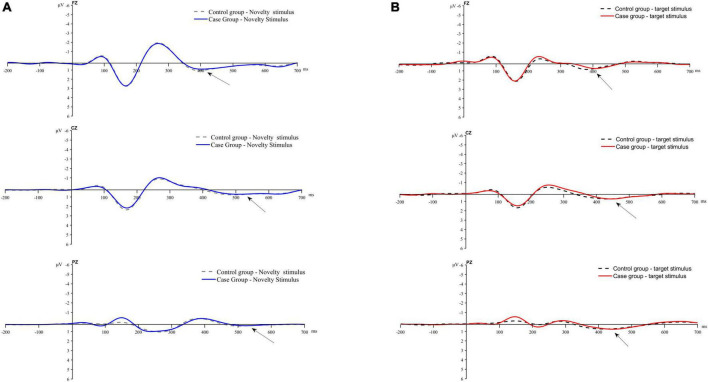
Average waveform plot. **(A)** Average waveform of P3a evoked by novel stimulis. **(B)** Average waveforms of P3b evoked by the target stimulus.

Figure 3 displays the average waveform graphs generated and recorded in the case and control groups under the two stimulation conditions at the FZ, CZ, and PZ electrodes.

#### 3.2.4 Results of dipole source reconstruction

As two patients in the control group exhibited a SNR of less than 3, significantly impacting the source reconstruction results, they were excluded. The final number of participants in the control group for source reconstruction was 146. In the study, source reconstruction analyses of the P3a and P3b components evoked by the novel and target stimuli were performed using Curry 7 software, respectively. The source reconstruction of the P3a component revealed that, compared to the control group, the case group exhibited significantly weaker current intensity in the right uncus–Brodmann area 20, the right superior temporal gyrus, the right middle temporal gyrus, the right subcallosal gyrus, and the right culmen (*p* < 0.05). Conversely, in the case group, compared to the control group, there was significantly stronger current intensity in the right precuneus, the left precuneus, the right precuneus–Brodmann 7 area, the right cingulate gyrus–Brodmann 31 area, the right cingulate gyrus, the left middle temporal gyrus–Brodmann 21 area, the left middle temporal gyrus, the left culmen, the right corpus callosum, and the left supramarginal gyrus–Brodmann 40 area (*p* < 0.05), as illustrated in Table 9.

**TABLE 9:** Brain regions with differences in activation intensity between the two groups of source reconstructions based on novel stimuli ($\bar{x}$ ± S, μAmm).

Activation of brain regions	Case group	Control group	*t*/*Z*	*P*
The right uncus–Brodmann area 20	32.16 ± 6.58	116.53 ± 72.03	−2.575*	0.028
The right superior temporal gyrus	9.10 ± 2.97	85.18 ± 74.72	−3.556	< 0.001
The right middle temporal gyrus	7.01 ± 2.42	17.28 ± 11.51	−2.468*	0.023
The right subcallosal gyrus	16.22 ± 17.77	43.6 ± 59.33	−3.939	< 0.001
The right culmen	26.11 ± 15.99	156.32 ± 60.05	−4.243	< 0.001
The right corpus callosum	111.26 ± 27.24	65.51 ± 33.98	−3.381	0.001
The right cingulate gyrus–Brodmann area 31	36.33 ± 14.69	21.6 ± 4.56	−2.245	0.025
The right cingulate gyrus	76.91 ± 35.46	37.07 ± 28.52	−3.839	< 0.001
The right precuneus–Brodmann area 7	10.18 ± 1.26	8.76 ± 0.65	−2.394	0.017
The right precuneus	11.51 ± 3.56	8.17 ± 0.49	−4.405	< 0.001
The left middle temporal gyrus-Brodmann area 21	4.89 ± 1.43	2.55 ± 0.37	−2.333	0.02
The left middle temporal gyrus	6 ± 0.86	4.74 ± 4.48	−3.141	0.002
The left culmen	108.8 ± 83.96	33.66 ± 11.69	3.213*	0.007
The left precuneus	15.18 ± 4.63	10.91 ± 3.68	−4.302	< 0.001
The left supramarginal gyrus -Brodmann area 40	3.47 ± 0.24	2.93 ± 0.39	−2.171	0.03

*denote *t*-test.

The source reconstruction of the P3b component indicated that, in comparison to the control group, the case group exhibited significantly weaker current intensity in the right lentiform nucleus–putamen, the right caudate–caudate head, the left subcallosal gyrus, the left inferior temporal gyrus, and the left temporal gyrus–Brodmann 20 (*p* < 0.05). Conversely, compared to the control group, the case group demonstrated significantly stronger activation intensity in the right superior temporal gyrus and the right middle temporal gyrus–Brodmann area 20 (*p* < 0.05), as detailed in Table 10.

**TABLE 10:** Brain regions with differences in activation intensity between the two groups of source reconstruction results based on target stimuli ($\bar{x}$ ± S, μAmm).

Activation of brain regions	Case group	Control group	*t*/*Z*	*P*
The right lentiform nucleus–putamen	38.4 ± 2.88	74.86 ± 23.24	−2.535	0.011
The right caudate–caudate head	32.46 ± 2.18	103.35 ± 20.72	−2.102	0.036
The left subcallosal gyrus	20.6 ± 16.69	182.29 ± 40.47	−2.049	0.04
The left inferior temporal gyrus	24.36 ± 13.07	200.64 ± 143.19	−2.701*	0.021
The left inferior temporal gyrus–Brodmann area 20	21.79 ± 12.77	125.39 ± 80.93	−3.662	0.000
The right superior temporal gyrus	85.37 ± 9.49	31.43 ± 19.37	−2.393	0.017
The right middle temporal gyrus–Brodmann area 20	73.88 ± 17.77	49.4 ± 31.9	−2.623	0.009

*denote *t*-test.

## 4 Discussion

### 4.1 Cognitive function in patients with multiple lacunar cerebral infarcts

The study population comprised patients with MLCI, and predominant infarct locations included the centrum semiovale, basal ganglia, paraventricular white matter, cerebellum, thalamus, and frontal lobe, all with NIHSS scores ≤ 4. Our prior research identified frontal lobe infarction as an independent risk factor for cognitive impairment development in patients with MLCI (Li et al., 2022). Furthermore, several studies have reported altered neurophysiological indices in MLCI patients. Tachibana et al. (1992, 1993) observed prolonged N200 and P300 latency in MLCI patients compared to the healthy population. Cao et al. (2017) reported prolonged P300 latency in MLCI patients compared to healthy populations. Additionally, Ma et al. (2004) found reduced P300 amplitude and prolonged latency in MLCI patients compared to healthy populations. Therefore, supported by evidence from multiple studies, cognitive dysfunction is observed in patients with MLCI.

### 4.2 Effect of stimulation conditions and electrode points on peak amplitude and peak latencies

In this investigation, the frontal lobe emerged as the predominant brain region activated in response to novel stimuli, specifically contributing to the mobilization of distractor attention capture resources in patients with MLCI. Our study revealed that individuals with MLCI exhibited reduced amplitude and prolonged latency for the P3a component associated with the novel stimulus compared to the P3b component related to the target stimulus, particularly in the central frontal, central, and central parietal regions. These findings are in line with prior research outcomes (Zhang et al., 2022). The P300 amplitude serves as an indicator of attentional resource allocation in the brain, while P300 latency correlates with the speed at which the brain processes external information, reflecting cognitive efficiency (Dallmer-Zerbe et al., 2020; Choinski et al., 2023). Diminished amplitude and prolonged latency signify challenges in allocating attentional resources and a deceleration in cognitive processing efficiency. Therefore, for patients with MLCI, the impairment in distractor attentional capture and cognitive processing efficiency was more pronounced in response to the P3a component of the novel stimulus compared to the task-related attentional and cognitive processing efficiency in response to the P3b component of the target stimulus. Additionally, our observations indicated that the P3a component exhibited the largest amplitude and shortest latency in the central frontal region, while displaying the smallest amplitude and longest latency in the central parietal region. Previous studies have proposed that P3a originates from stimulus-driven frontal attentional mechanisms during task processing (Polich, 2007). Despite more severe impairments in distracted attention capture resources and cognitive processing efficiency observed in patients with MLCI, the frontal lobe retained its primary role in mobilizing functions related to the capture of distracted attention resources in this specific patient population.

### 4.3 Analysis of visual P3a and P3b peak amplitude and peak latency results

Studies have indicated that the frontoparietal lobe plays a crucial role in regulating attentional resources (Zhang et al., 2022), and there is functional connectivity between the cerebellum and the frontoparietal lobe (Ruan et al., 2023). Reductions in P300 amplitude have been observed in patients with cerebellar injury (Rodriguez-Labrada et al., 2019; Nanda et al., 2023), suggesting the cerebellum’s involvement in attentional resource allocation. Another study linked the early reduction in P3b wave amplitude following cerebellar infarction to dysfunction in cerebellar projections to the prefrontal and posterior parietal cortex (Mannarelli et al., 2015). Numerous fiber bundles converge in the center of the semiovale, the basal ganglia region, and the paraventricular white matter of the lateral ventricles, connecting the frontoparietal lobes with the cerebellar cortex. P300 is significantly associated with cerebral white matter damage and correlates with the severity of cognitive impairment (Nei et al., 2022). In our study, prevalent infarction locations included the centrum semiovale, paraventricular white matter of the lateral ventricles, and the basal ganglia region. This can lead to impaired fiber conduction between the frontoparietal lobes and the cerebellum, causing the cerebellum’s function of allocating attentional resources to malfunction. This is supported by the observation that the peak amplitude did not show a statistically significant difference between the two groups in this study. The amplitude of the P3a component is linked to distractor attention capture resource allocation, while the amplitude of the P3b component is associated with task-related attention resource allocation. Therefore, we conclude that the site of brain injury in our study affected the cerebellum’s function in coordinating the allocation of attentional resources. The cerebellum was unable to compensate for the resource mobilization of the brain’s visually selective attentional function, regardless of the presence or absence of cerebellar infarction.

P300 Latency represents the time taken by the brain to recognize a stimulus after cognitive processing from perceiving the stimulus, reflecting the speed at which the brain encodes, classifies, and recognizes the stimulus. Prolonged latency suggests a slowing down of information processing, and cognitive dysfunction is dominated by prolonged latency (Guo et al., 2022). In the present study, we observed a significant prolongation in the latency of the P3a and P3b components in the group of cases with cerebellar infarction compared to the control group without cerebellar infarction. Novel stimuli trigger transient attentional capture and persistent increases in phasic arousal (Masson and Bidet-Caulet, 2019). Prolonged P3a latency suggests that cerebellar infarction leads to alert attention dysfunction in MLCI patients. Target stimuli trigger task-related sustained attentional processing, and prolonged P3b latency suggests that cerebellar infarction leads to impaired sustained attentional function in MLCI patients. It has been suggested that the significant prolongation of P300 latency in stroke patients is due to cerebral cortical damage, cerebral neurological structural and functional disorders, localized inflammatory responses, and metabolic abnormalities in brain tissues, as well as imbalances in the production of neurotransmitters (Guo et al., 2022). The “internal model” hypothesis suggests that the cerebellum can reproduce cortical psychology by encoding internal models (Ito, 2008). Additionally, research has demonstrated that the cerebellum plays a role in decision-making calculations (Deverett et al., 2018), and studies have found that patients with cerebellar injury are more likely to make decision-making errors (Lai et al., 2023; van der Giessen et al., 2023). Another study concluded that the cerebellum is not directly involved in attentional functions and likely plays a role by coordinating and managing levels of activation or inhibition in cortical areas (Mannarelli et al., 2016).

Patients with MLCI often experience symptoms such as dizziness, headaches, and various cognitive dysfunctions, including issues with memory and attention. There is a heightened risk of developing vascular dementia in the long term. Visual selective attention function is impacted by interference from both target stimuli and novel stimuli. Selective information processing bias may contribute to the development and persistence of anxiety disorders (Valadez et al., 2022). Previous research has identified attentional bias as a crucial factor in the progression from early-life stress to depression (Mao et al., 2020). The cognitive dysfunction observed in these patients can be accompanied by emotional disturbances, significantly impacting their overall physical and mental health and wellbeing. ERPs allow for the early detection of subclinical visual selective attention dysfunction, facilitating early intervention and treatment. They provide an effective and objective basis for neurocognitive rehabilitation. The modulation of the cerebellum through physical stimulation, such as transcranial direct current stimulation (Mannarelli et al., 2019), has shown promise in improving visual selective attention function. This improvement can help mitigate the negative impact of anxiety and depression resulting from visual selective attention dysfunction on daily life, work, and neurological rehabilitation.

### 4.4 Source reconstruction analyses

Novel stimuli activate bottom-up sensory stimulus-related ventral frontoparietal attentional networks, primarily situated in the temporoparietal junction and ventral frontal cortex of the right hemisphere (Corbetta and Shulman, 2002; Vossel et al., 2014). We observed that when novel stimuli were presented, the current intensity in the case group with cerebellar infarction was significantly weakened in the right uncus–Brodmann area 20, the right superior temporal gyrus, the right middle temporal gyrus, the right subcallosal gyrus, and the right culmen compared with the control group. The uncus is a crucial component of the medial temporal lobe, and its posterior portion constitutes the head of the hippocampus (Patra et al., 2018; Fogwe et al., 2023). Patients with injury to the medial temporal lobe often experience a decline in working memory (Goodrich and Yonelinas, 2016). The hippocampus and the adjacent medial temporal lobe play a role in processing novel thoughts, and the middle temporal gyrus enhances this function in the hippocampus (Ren et al., 2020). The right superior temporal gyrus is involved in stimulus-centered ectopic spatial processing (Shah-Basak et al., 2018). Decreased blood flow in the right subcallosal gyrus is associated with amnesia (Mugikura et al., 2020). Reduced culmen gray matter volume is associated with attentional dysfunction (Duan et al., 2018). Therefore, when MLCI patients with cerebellar infarction viewed novel stimuli, the uncus and middle temporal gyrus involved in novelty processing, the right superior temporal gyrus involved in visuospatial processing, the right inferior corpus callosum gyrus involved in memory-related activation, as well as the culmen involved in attentional functions, and the visual distraction of the attention-capturing and resource-allocating functions of the cerebellar infarcted patients with MLCI were weakened. In addition, compared to the control group, the case group with cerebellar infarction had significantly increased current intensity in the right precuneus, the left precuneus, the right precuneus-Brodmann 7, the right cingulate gyrus-Brodmann 31, the right cingulate gyrus, the left middle temporal gyrus-Brodmann 21, the left middle temporal gyrus, the left culmen, the right corpus callosum, and the left supramarginal gyrus–Brodmann 40. We hypothesize two reasons for the increased activation in these brain regions: firstly, as a compensation for reduced activation in certain aforementioned brain regions, one side of the brain exhibits decreased activation while the corresponding region on the other side shows increased activation (e.g., decreased activation of the right culmen and increased activation of the left culmen, decreased activation of the right middle temporal gyrus and increased activation of the left middle temporal gyrus); secondly, to compensate for the bottom-up sensory stimulus-driven ventral frontoparietal attentional network, specifically the temporoparietal junction and ventral frontal cortex on the right side of the brain. Anatomically, the right corpus callosum, right cingulate gyrus, and right precuneus are all located in the medial aspect of the right brain and are arranged in a bottom-up manner, aligning with the activation sequence of the right ventral frontoparietal network driven by bottom-up sensory stimulation, with the left supramarginal gyrus corresponding to the right temporoparietal junction. Therefore, we hypothesize that cerebellar infarction leads to enhanced activation of the above brain regions in order to compensate for ventral frontoparietal attention network function. Functionally, the precuneus belongs to the association cortex and is extensively connected to other cortical and subcortical structures, and visual stimuli attentional shifts cause bilateral precuneus activation (Cavanna and Trimble, 2006). Both the right cingulate gyrus and the right subcallosal gyrus belong to the right limbic system, which has attentional, memory, and emotional functions (Sitoh and Tien, 1997). In patients with cerebellar infarction who mobilize distractor functions, diminished activation of the right inferior corpus callosum gyrus leads to enhanced compensatory activation of the right cingulate gyrus, which conduct excitation directly to the right ventral frontal lobe. The findings of this study indicate that individuals with MLCI, particularly those with cerebellar infarction, may potentially enhance compensation for bottom-up sensory stimulation related to the ventral frontoparietal attentional network by influencing the excitability of the aforementioned brain regions.

Target-directed stimuli drive a top-down dorsal frontoparietal attentional network that primarily activates the posterior parietal and frontal lobes of the brain bilaterally (Corbetta and Shulman, 2002; Vossel et al., 2014; Breu et al., 2023). Breu et al. (2023) found hyperexcitability of frontoparietal network brain regions when vision follows volitional control, including the dorsolateral brain prefrontal ependymal cortex, the base of the prefrontal brain region, the insula of the forebrain, the precuneus, and the posterior parietal cortex. Previous studies have found significant reductions in frontal and temporal gray matter, caudate nucleus and cerebellar volume in children with attention/hyperactivity disorder, and a positive correlation with severity (Castellanos et al., 2002). We found that the intensity of activation was significantly weaker in the case group with cerebellar infarction compared with the control group in the right lentiform nucleus–putamen, the right caudate–caudate head, the left subcallosal gyrus, the left inferior temporal gyrus, and the left inferior temporal gyrus-Brodmann area 20. The right lentiform nucleus–putamen and the right caudate–caudate head belong to the right basal ganglia region. Impairment of the function of the basal ganglia as a relay station connecting cortical and subcortical structures leads to attentional dysfunction (Riva et al., 2018). It has been found that distal brain atrophy and prefrontal lobe functional dissociation are important causes of cognitive impairment in patients with basal ganglia infarction (Jia et al., 2024). The left subcallosal gyrus belongs to the limbic system and has an attentional function (Sitoh and Tien, 1997). The inferior temporal gyrus plays a role in visual object recognition, decision making, and is connected to both the frontal and parietal lobes (Lin et al., 2020). The case group with cerebellar infarction showed significantly stronger activation in the right superior temporal gyrus and the right middle temporal gyrus-Brodmann 20 region compared with the control group. We hypothesize that diminished activation of the left inferior temporal gyrus leads to enhanced compensatory activation of the right superior temporal gyrus and middle temporal gyrus. Therefore, the findings of the current study imply that MLCI patients with cerebellar infarction, through modulating the excitability of the mentioned brain regions, aim to impact the top-down dorsal frontoparietal attentional network directed by goal-directed stimuli.

## 5 Limitations of the research

We conducted a case-control study to investigate the electrophysiological characteristics of the cerebellum in relation to visually selective attention functions and neural network mechanisms. Our future plans include incorporating healthy controls to enhance the comprehensiveness of our findings. Additionally, this study is currently conducted at a single center, and we intend to expand to a multi-center study in the next phase to augment the sample size. Lastly, the source reconstruction method employed in this study necessitates EEG data with high SNR, imposing increased demands on the acquisition of EEG data information.

## 6 Conclusion

In our current investigation, we observed that individuals with MLCI exhibited more pronounced deficits in distracted attention capture. Notably, the frontal lobe persisted as the primary brain region orchestrating the mechanisms associated with distracted attention capture. Moreover, our findings underscore the significant role of the cerebellum in governing the allocation of visual selective attentional resources among individuals with MLCI. MLCI patients with cerebellar infarction may have attempted to compensate for the visually selective attentional function of MLCI patients by modulating connections to the aforementioned cerebral brain regions. However, the cerebellum exhibited a lack of compensation, as evidenced by the absence of changes in P300 amplitude and the significant prolongation of latency in the cerebellar infarction case group compared with the control group. Therefore, the cerebellum indirectly influences the ventral and dorsal frontoparietal attention networks by modulating the levels of excitation and inhibition within the cerebral cortex of the attention network. This mechanism may represent a potential pathway through which the cerebellum regulates visual selective attention information in MLCI patients.

## Data availability statement

The raw data supporting the conclusions of this article will be made available by the authors, without undue reservation.

## Ethics statement

The studies involving humans were approved by the Kailuan General Hospital Disciplinary Committee. The studies were conducted in accordance with the local legislation and institutional requirements. The participants provided their written informed consent to participate in this study. Written informed consent was obtained from the individual(s) for the publication of any potentially identifiable images or data included in this article.

## Author contributions

XY: Project administration, Writing – review & editing. LD: Data curation, Investigation, Methodology, Software, Writing – original draft, Writing – review & editing. YO: Writing – review & editing. QL: Investigation, Writing – review & editing. JW: Writing – review & editing. JZ: Writing – review & editing. LC: Writing – review & editing. HQ: Writing – review & editing. PZ: Funding acquisition, Project administration, Writing – review & editing.
